# Individual and combined effects of amoxicillin and carbamazepine to the marine copepod *Tigriopus fulvus*

**DOI:** 10.1007/s11356-023-26498-0

**Published:** 2023-03-18

**Authors:** Ermelinda Prato, Francesca Biandolino, Asia Grattagliano, Andrea Ruscito, Giusy Lofrano, Giovanni Libralato, Marco Trifuoggi, Luisa Albarano, Isabella Parlapiano

**Affiliations:** 1 National Research Council, Water Research Institute (IRSA-CNR), Via Roma, 3, 74123 Taranto, Italy; 2 Department of Chemical Sciences and Technologies, University of Rome “Tor Vergata”, Via Della Ricerca Scientifica, 1 – 00133 Rome, Italy; 3 Università degli Studi di Roma Foro Italico, Piazza Lauro De Bosis, 15, 00135 Rome, Italy; 4 Department of Biology, University of Naples Federico II, Via Vicinale Cupa Cintia 26, 80126 Naples, Italy; 5 Department of Chemical Sciences, University of Naples Federico II, Via Vicinale Cupa Cintia 26, 80126 Naples, Italy

**Keywords:** Amoxicillin, Carbamazepine, Mixture, *Tigriopus fulvus*, Acute and chronic toxicity

## Abstract

Pharmaceuticals can be considered a global threat to aquatic ecosystems due to their pseudo-persistence and their potential toxicity towards non-target species. Amoxicillin (AMX) and carbamazepine (CBZ) and their mixture (1:1) were investigated on the marine copepod *Tigriopus fulvus* (Fischer, 1860) considering both acute and chronic endpoints. While acute and chronic exposure did not directly affect survival, reproductive endpoints were affected like the mean egg hatching time that was significantly longer than the negative control for treatments with AMX (0.789 ± 0.079 μg/L), CBZ (8.88 ± 0.89 μg/L), and AMX and CMZ as a mixture (1.03 ± 0.10 μg/L and 0.941 ± 0.094 μg/L), in that order.

**Supplementary Information:**

The online version contains supplementary material available at 10.1007/s11356-023-26498-0.

## Introduction

The widespread use of pharmaceuticals improved the general quality of life increasing life expectancy as well. Despite these benefits, the chemical and (eco)toxicological studies have raised an increasing concern over the potential threats of pharmaceuticals to both the aquatic environment and human health (Kurunthachalam 2012). The European Commission (Deloitte Sustainability 2018) and HELCOM (International Initiative on Water Quality-IIWQ 2017) declared pharmaceuticals as emerging contaminants (ECs).

Pharmaceuticals can enter in the aquatic environment through different pathways: discharge of wastewater from domestic households, industrial effluents, agricultural effluents, aquaculture, and solid wastes. Several studies have shown that numerous pharmaceuticals are discharged into water bodies (Corcoran et al. 2010; Mutiyar and Mittal 2014; Chen et al. 2015; Mezzelani et al. 2018). The concentrations of these products can significantly differ amongst countries depending on the consumption and population. Their presence in surface waters, groundwater, and even marine systems have been estimated at ng/L or µg/L concentrations (Tran et al. 2017). Moreover, wastewater treatment plants (WWTPs) often are not able to remove drugs, favouring their introduction into the aquatic ecosystem (Verlicchi et al. 2012; Zhang et al. 2018).

This suggests the need to address potential exposure scenarios that could trigger toxic effects on non-target organisms (Richardson et al. 2005; Claessens et al. 2013). To date, the knowledge about the toxicological effects of pharmaceuticals in the aquatic environment must be strengthened especially for saltwater species (Chen et al 2019a; Siciliano et al. 2021).

The present paper focused on the antibiotic amoxicillin (AMX) and the antiepileptic carbamazepine (CBZ) (Jones et al. 2002; Lalumera et al. 2004; da Silva Santos et al. 2018; Mezzelani et al. 2018, 2020), being the two most frequently detected pharmaceuticals in the aquatic environment. Antibiotics are widely used both for treatment of human and animal diseases. After their administration, they are not fully metabolized so they are discharged from the body both in feces and urines. Studies have demonstrated that low concentrations of antibiotics can accelerate the evolution of antibiotic-resistant bacteria and antibiotic resistance genes, with adverse health problems to humans (Liu et al. 2018; Ramesh et al. 2018). In Europe, Amoxicillin (AMX) is among the most prescribed antibiotics for both human and animal use (Lalumera et al. 2004; Jones et al. 2002), including aquaculture (Siciliano et al. 2021). Although they are usually measured at trace concentrations (i.e., ng/L to µg/L in water and µg/kg to mg/kg in soil/sediment), AMX is a “pseudo-persistent” contaminant due to its constant use and release (Daughton and Ternes 1999; Hernando et al. 2006).

Carbamazepine (CBZ) is an anticonvulsant drug used for the treatment of epilepsy, bipolar disorder, and trigeminal neuralgia (Calcagno et al. 2016). Pomati et al. (2006), Qiang et al. (2016), and Verlicchi et al. (2012) reported ng/L of CBZ in surface water samples, while Mezzelani et al. (2020) observed the presence of 35 ng/g dry weight (d.w.) up to 280 ng/g d.w. of CBZ in tissues of aquatic invertebrates. CBZ is not fully metabolized by humans, and only partially removed in wastewater treatment plants (WWTPs) (< 10%) persisting into the environment (Contardo-Jara et al. 2011).

Marine coastal areas are traditionally impacted by contaminants from rivers, streams, and wastewater effluents (Martínez et al. 2007; Fernández et al. 2016; Fernández-Rubio et al. 2019). To date, much of the research focused on the acute toxicity of pharmaceuticals to freshwater species (Liu et al. 2015; Miller et al. 2019), neglecting their acute and chronic effects on marine organisms (of individual pharmaceuticals and mixtures) (Arnold et al. 2014; Gaw et al. 2014; Rodríguez-Mozaz et al. 2017; Franzellitti et al. 2019; Mezzelani et al. 2016; Trombini et al. 2016).

Among invertebrate copepods, *Tigriopus fulvus* (Fischer 1860) is used as testing species in bioassays because of its suitability for laboratory rearing (Faraponova et al. 2005), good sensitivity to different toxicants, and data reproducibility (Faraponova et al. 2005, 2016; Mariani et al. 2006; Tornambè et al. 2012; Prato et al. 2011, 2012, 2013, 2015, 2019; Biandolino et al. 2018). This species represents an important link in the marine food chain since it feeds on microalgae or bacteria, and it is a prey for larger crustaceans, fish larvae, and filter-feeding bivalves.

The present study evaluated the acute and chronic toxicity of amoxicillin and carbamazepine as pure substances and in mixture (1:1) to the marine copepod *T. fulvus* including a multi-endpoint approach (survival, growth, and reproduction).

## Materials and methods

### Experimental animals

The harpacticoid copepod *T. fulvus*, originally obtained from cultures coming from the Northern Tyrrhenian Sea, has been reared for multiple generations at National Research Council, Institute for Water Research (CNR-IRSA) in Taranto (Italy). The culture was maintained in natural seawater (NSW, filtered 0.45 μm through cellulose membranes; salinity 38 psu) in a thermostatic chamber at 20 ± 1 °C with a 16:8 h L/D photoperiod. *Tigriopus fulvus* was fed twice a week using a mixed algal diet: *Tetraselmis suecica* and *Isochrysis galbana* at 1.5 × 10^8^ and 3.0 × 10^8^ cells/L, respectively.

Toxicity tests were carried out on newborn offspring (nauplii) originating from synchronized cultures (24–48 h) enabling the use of the same developmental stage. To obtain the synchronized nauplii, about 200 females with egg sacs were collected from the stock culture and transferred to an 80 μm mesh plankton net fixed on a Plexiglas tube placed in a Petri dish, to allow the passage of newly hatched nauplii. After 24 h, healthy nauplii (i.e., able to actively swim) were randomly selected with a Pasteur pipette under a stereomicroscope, washed by gently pipetting them in clean artificial saltwater (ASW, filtered at 0.45 μm), and transferred in sterile 12 multi-well plates (5 mL per well) (Nest Biotech Co., Ltd).

### Exposure media preparation

The chemicals and reagents used in this study were purchased from Sigma-Aldrich (Zwijndrecht, the Netherlands) and were of analytical grade. Amoxicillin trihydrate (CAS 61336–70-7, purity > 99%) and Carbamazepine (CAS 298–46–4, purity > 97%) were dissolved in methanol high-performance liquid chromatography (HPLC) grade (purity ≥ 99.9) to prepare concentrated stock solutions (1000 mg/L). These solutions were stored at 4 °C in amber glass vials for no longer than 2 weeks to minimize photodegradation.

### Ecotoxicity

The experimental design of this study was devoted to (i) determining the acute toxicity of carbamazepine and amoxicillin as pure substances and their 1:1 mixture, (ii) and evaluate their chronic effects with sublethal endpoints using *T. fulvus*. The exposure solutions for individual acute tests of AMX and CBZ were prepared as follows: 6.25, 12.5, 25.0, 50.0, and 100 mg/L (nominal concentrations). The highest tested concentration was 100 mg/L because according to the EC-Directive 93/67/EEC (European Commission 1993), substances with EC50 values higher than 100 mg/L are not considered harmful to aquatic organisms. The binary mixtures of the two pharmaceuticals were in a ratio of 1:1.

The individual and combined chronic toxicities of AMX and CBZ were examined. To simulate natural conditions in aquatic ecosystems and plausible environmental worst-cases scenarios, chronic tests were conducted exposing copepods to a wide range of concentrations with an increasing factor of tenfold. The nominal investigated concentrations were: 0.1, 1, 10, and 100 μg/L (for both pure substance and the mixture). ASW was used as a negative control and to prepare testing solutions. Copper sulphate (CuSO_4_ × 5H_2_O, 0.015, 0.03, 0.06, 0.12, 0.25, 0.50, 1.00 mg/L of Cu^2+^) was used as positive control ensuring the validity of the test (UNICHIM 2396:2014, 2014) (Faraponova et al. 2016). The experimental conditions of acute and chronic tests are summarised in Table S1 (Supplementary Materials).

#### Acute exposure test (96 h)

Acute tests of naupliar mortality were performed according to ISO (1999) and the modifications introduced by Prato et al. (2013). Briefly, triplicate groups of ten nauplii (≤ 24 h old) were transferred in 12-multiwell plates filled with 3 mL of experimental concentrations: ASW (negative control), copper (positive control), AMX, CBZ and their mixture. Tests solutions were renewed after 48 h. No food was supplied during the entire duration of the exposure. The mortality of copepods was assessed after 96 h of exposure, by inspecting the wells under a stereomicroscope. Nauplii were considered dead if they did not actively swim after 20 s of observation and light stimulation.

#### Chronic exposure (28 days)

The individual chronic toxicities of AMX and CBZ and their binary mixtures (1:1) to the copepods was investigated with a full life-cycle approach (Kwok et al. 2009). Briefly, triplicate groups of 12–13 nauplii (< 24 h) per treatment were randomly selected and transferred to 12-well culture plates containing 4 mL of test solution. Spiked test media supplemented with *T. suecica* (10^5^ cells/mL) were renewed (> 80% of the working volume) every 2 days. Wells were checked daily under a stereomicroscope to record mortality and developmental stages until copepods reached the adult stage. The males were discarded after mating, and the experiment was continued with ovigerous females only. To measure reproductive endpoints seven ovigerous females per treatment were individually transferred to a new 12-well culture plate in a volume of 2 ml of test solution until the offspring were released. Each well was observed daily with a renewal every 48 h when the females were transferred to a new culture plate with fresh solutions; hatched nauplii were counted under a stereomicroscope. In total, 8 life cycle traits were examined: lethality, nauplii percentage that reached copepodite stage after 5 days, development time to maturation of females (i.e., development of the egg sac), sex ratio, hatching time, mean brood per female, mean number of nauplii per brood female and aborted egg sacs.

### Chemical analysis

Artificial saltwater (Instant Ocean®, pH 8.0 ± 0.1, Salinity, 38 ± 2 psu, filtered through a GF/C Whatman 1.2 μm mesh) was used as dilution water. Before adding ASW, methanol was completely evaporated under a gentle stream of nitrogen. Three samples per testing concentrations were collected prior to toxicity testing and processed as follows. Each sample was extracted by solid-phase extraction (SPE), 0.5 L of the sample was filtered and pre-concentrated on cartridges made of polystyrene-divinylbenzene resin (STRATA XL 6 mL/500 mg—Phenomenex). The cartridges were preconditioned with methanol and then distilled water. The analytes were eluted with a solution of 1–5 mL of methanol/acetonitrile (1:1). The extract was then concentrated to 0.1 mL under nitrogen flow (Multivap8, LabTech, Italy). The extract was injected into an HPLC system consisting of a 20AD XR LC pump, a SIL 2A HT autosampler, and a DAD SPD M20A UV detector (All Shimadzu, Japan). HPLC separations were performed on a 150 mm × 4.6 mm, 5 µm C18 column (Phenomenex, USA). The mobile phase consisted of a binary mixture of solvents: (A) 95% of ammonium acetate solution at pH 4.0 and (B) 5% acetonitrile. Separations were performed at room temperature, and the flow rate was maintained at 1 mL/min. The compounds were monitored at a wavelength of 254 nm. The detection limit (LOD) and limit of quantification (LOQ) (ICH, 2005) were 0.002 μg/L and 0.006 μg/L.

### Statistical analyses

Tests were performed in triplicate, repeated on three distinct occasions, and statistical analyses were completed using Statgraphics software and package software Past3 (version 1.0). For acute toxicity tests, the 96 h LC50 values, were calculated using the Spearman-Karber method (USEPA 1994—ToxStat software package). No observed and lowest observed effect concentrations (NOECs and LOECs) were calculated for all endpoints using analysis of variance (ANOVA) from the observed data. Maximum acceptable toxicant concentration (MATC) was calculated as the geometric mean of NOEC and LOEC values. Analysis of variance (ANOVA) was applied for all the analysed parameters to test differences among treatments and between all treatment concentrations. Raw data were tested for normality and homogeneity of variances using Shapiro-Wilks and Bartlett’s tests. Both assumptions were met, data were examined by analysis of variance (one-way ANOVA) and a multiple comparison procedure (Tukey test) to find significant variations (*p* < 0.05) among treatments. When requirements for normality and homogeneity were not met, the non-parametric Kruskal–Wallis test on ranks was applied followed by Dunn’s post hoc test. The level of significance was always set at α = 0.05. Whenever necessary, nested ANOVA was considered to verify if between-runs variance did not differ before pooling the data.

## Results

### Acute toxicity test

The mean percentage of survival in the negative controls was > 90% in each experiment, meeting the acceptability criteria established for the test (Faraponova et al. 2016). The median lethal concentration (LC_50_) of the positive control was equal to 0.11 (0.08–0.16; nominal concentration) mg/L of Cu^2+^ being in accordance with the reference guideline (UNICHIM 2396: 2014, 2014).

After 96 h of exposure, the two drugs tested individually and in mixture slightly affected survival of *T. fulvus*. The highest mortality rate was equal to 44% in nauplii exposed to CBZ (100 µg/L) and 22% in those exposed to MIX (100 + 100 µg/L). For AMX, no effect was evidenced even at 100 mg/L (nominal concentration). Therefore, LC_50_ values were not determined at the investigated concentrations.

### Chronic test

#### Chemical data

Measured concentrations for AMX, CBZ, and their mixture are summarized in Table 1 including both nominal and measured values used in chronic toxicity tests.

**Table 1:** Measured concentrations (media ± SD) of carbamazepine (CBZ) and amoxicillin (AMX) in chronic toxicity tests with single compounds and a mixture (1:1). The data are reported in µg/L (*n* = 9)

Nominal concentrations		Measured concentrations	
Experimental design		Amoxicillin	Carbamazepine
Istant Ocean™ (38 psu)	-	< 0.002	< 0.002
AMX	0.1	0.080 ± 0.005	-
	1	0.789 ± 0.042	-
	10	9.540 ± 0.416	-
	100	93.40 ± 3.79	-
CBZ	0.1	-	0.16 ± 0.01
	1	-	1.26 ± 0.08
	10	-	8.88 ± 0.41
	100	-	95.60 ± 3.98
AMX + CBZ	0.1 + 0.1	0.076 ± 0.005	0.084 ± 0.005
	1 + 1	0.941 ± 0.059	1.030 ± 0.065
	10 + 10	9.214 ± 0.402	8.741 ± 0.399
	100 + 100	96.34 ± 2.47	94.67 ± 2.97

#### Toxicity data

**Fig. 1:**
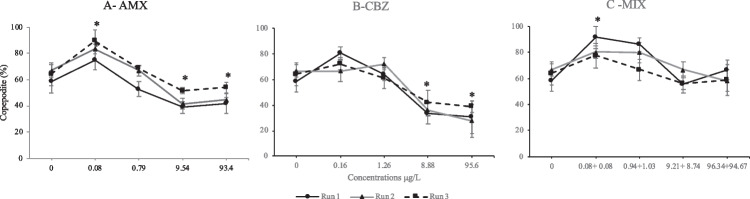
Percentage (%) of *T. fulvus* larval development (from nauplii to copepodites) during the first 5 days after the exposure to **A** amoxicillin (AMX), **B** carbamazepine (CBZ), and **C** their mixture (MIX). Concentrations are in μg/L; effect data are reported as mean ± SD (*n* = 3) of three runs, each replicated three times (*n* = 9), Tukey’s test (*p < 0.05)

After 28 days of exposure, all copepods tested showed good survival percentages (> 95%) for all treatments without significant differences from the control (*p* > 0.05, data not shown). Data about the percentage of developed copepodite after 5 days are highlighted in Fig. 1 A, B, and C. During the first 5 days of exposure, the mean percentage (%) of larval development (from nauplii to copepodites) in the negative controls was 63 ± 4% (Fig. 1). A significant increase in copepodites percentage was observed at 0.080 μg/L, while a lower percentage of developed copepodites exposed to AMX was shown at 9.540 μg/L and 93.40 μg/L (ANOVA, *F*-ratio = 16, *p* < 0.05) (Fig. 1A). A significant inhibition of the larval development was observed at 8.88 μg/L and 95.6 μg/L of CBZ (ANOVA, *F*-ratio = 0.53, *p* < 0.05) with 41% and 37% of nauplii developed to the copepodite stage, respectively (Fig. 1B). The MIX samples caused a significant increase of larval development at 0.08 + 0.75 μg/L of CBZ + AMX (ANOVA, *F*-ratio = 7.14, *p* < 0.05), while no effect was observed at the highest concentrations after 5 days of exposure (Fig. 1C). The MATC values were 2.74, 3.34, and 0.05 + 0.06 μg/L for AMX, CBZ, and MIX, respectively (Table 2).

**Table 2:** Maximum acceptable tolerance concentration (MATC) of amoxicillin (AMX), carbamazepine (CBZ), and their mixture 1:1 (MIX) on various endpoints

		MATC (μg/L)	
	AMX	CBZ	MIX
Larval development	2.74	3.34	0.05 + 0.06
Hacthing time	0.25	3.34	0.27 + 0.29
Brood per female	0.25	0.11	0.27 + 0.29
Nauplii per brood	> 100	> 100	> 100
Nauplii per female	2.74	> 100	0.27 + 0.29
Aborted sacs	29.8	29.1	2.94 + 3.00

Amoxicillin, CBZ and their MIX did not significantly slow down larval development of *T. fulvus* after 5 days of exposure as shown in Supplementary Materials (Table S2). Indeed, the apparent differences between runs are related to the intrinsic variability within replicates as suggested by the results of the nested analysis of variance displayed in Supplementary Materials (Table S3).

There were no significant differences in sex ratios, which varied between 0.8 and 1.4 (data not shown).

**Fig. 2:**
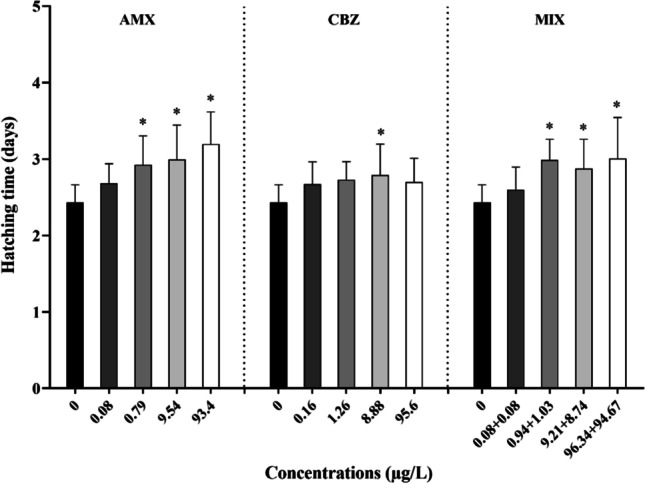
Hatching time (days) of *T. fulvus* exposed to amoxicillin (AMX), carbamazepine (CBZ), and their mixture 1:1 (MIX). Concentrations are in μg/L and effect data are reported as mean ± SD (*n* = 9), Tukey’s test (*p < 0.05)

The time required for the release of the offspring was 2.4 ± 0.2 days in the negative controls. A significant concentration dependent increase in hatching time was observed starting from 0.79 μg/L of AMX (*F* = 9.50; *p* < 0.05; Fig. 2), with a MATC value of 0.25 μg/L (Table 2). In particular, the offspring releases occurred after 3.2 ± 0.4 days at the highest concentration of AMX (100 μg/L). CBZ showed significant differences only at 8.88 μg/L compared to the control and all tested concentrations (*F* = 2.86, *p* < 0.05) and a MATC value of 3.34 μg/L (Table 2). Mixtures displayed a significant increase of time nauplii release starting from 0.94 + 1.03 μg/L of CBZ + AMX (*F* = 6.90, *p* < 0.05; Fig. 2) and MATC value of 0.27 and 0.29 μg/L for AMX and CBZ (Table 2), respectively. The hatching time data of each run test (*n* = 3) are summarized in Table S4.

**Fig. 3:**
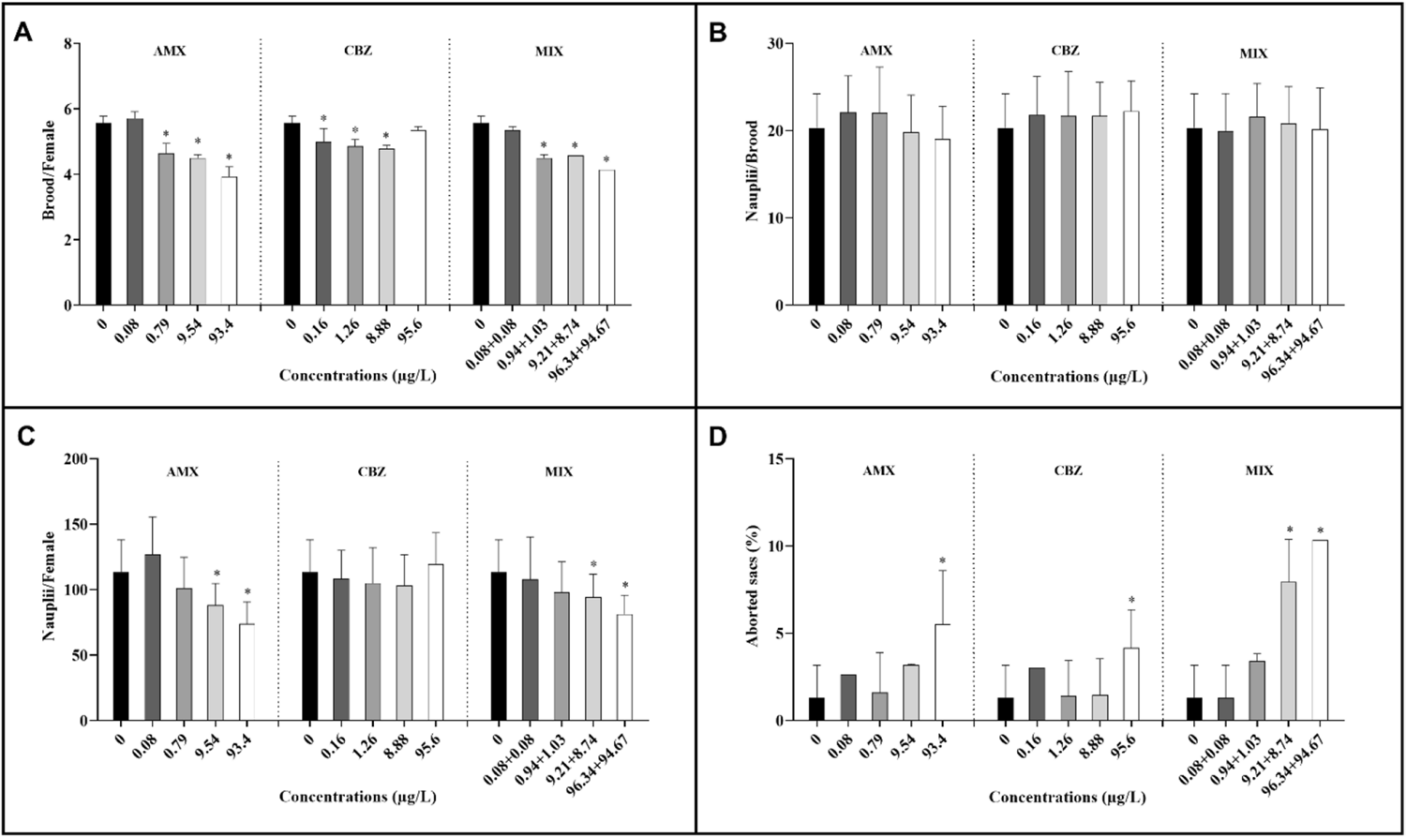
Effect of Carbamazepine (CBZ), amoxicillin (AMX), and their mixture 1:1 (MIX) on some reproductive traits: **A** number of broods per female; **B** number of nauplii per brood and **C** per female; and **D** aborted sacs. Effect data was reported as mean ± SD (*n* = 9), Tukey’s test (*p < 0.05) compared to the negative control

As reported in Fig. 3, chronic exposure to AMX led to a significant reduction in the mean number of broods per female compared to the control at the tested concentrations over 0.080 ± 0.005 μg/L (*F* = 21.6, *p* < 0.05), with a MATC value of 0.25 μg/L (Table 2). The number of broods ranged from 5.6 ± 0.5 in the control to 3.9 ± 0.7 at 93.40 ± 9.34 μg/L (Fig. 3A).

A significant decrease in the number of broods was also observed in CBZ treatments at 0.16, 1.26, and 8.88 μg/L (*F* = 12.9, *p* < 0.05), while at 95.60 ± 9.56 μg/L, the number of broods was almost equal to the control (Fig. 3A). The calculated MATC value was equal to 0.11 μg/L (Table 2). The exposure to the MIX showed a significant decrease starting from 1.030 to 0.941 μg/L of AMX and CBZ (*F* = 161, *p* < 0.05), respectively. The mean number of broods per female at the highest concentration was 4.1 ± 0.8 (Fig. 3A). AMX, CBZ, and MIX did not affect the average number of nauplii produced per brood (*p* > 0.05; Fig. 3B).

At the end of the experiment, the total number of nauplii per female in the control was 113.5 ± 24.6 (Fig. 3C). A significant decrease of nauplii per female was observed for AMX treatment at 9.540 µg/L and 93.40 µg/L (88.1 and 73.9, respectively) (*F* = 11.84, *p* < 0.05) and for MIX starting from 0.94 + 1.03 µg/L of AMX + CBX (*F* = 4.03, *p* < 0.05; Fig. 3C). The calculated MATC value for AMX was equal to 2.74 μg/L (Table 2).

Reproductive failure, defined as the percent of broods per females unable to produce viable offspring (aborted egg sacs) significantly increased at the maximum tested concentration of AMX and CBZ, (*F* = 7.3 and 4, respectively; *p* < 0.05) compared with lower exposures and controls (*p* < 0.05), while exposure to the MIX showed a significant increase at 9.2 + 8.7 and 96.3 + 94.7 µg/L of AMX + CBX (*F* = 61.7, *p* < 0.05; Fig. 3D). The estimated MATC values were 29.8 and 29.1 for AMX and CBZ, in that order (Table 2).

The effect of carbamazepine (CBZ), amoxicillin (AMX), and their mixture 1:1 (MIX) on number of broods per female; number of nauplii per brood and per female; and aborted sacs data of each run test (*N* = 3) are summarized in Tables S5–S6 (Supplementary Materials).

## Discussion

AMX is a bactericide capable of inhibiting certain enzymes responsible for the synthesis of the cell walls of bacteria, determining cell lysis (Kaur et al. 2011), while the effects triggered by CBZ on marine invertebrates exposed to a range of environmentally realistic concentrations of CBZ (0.3–3.0 and 6.0–9.0 μg/L), showed alterations of the oxidative status, lipid peroxidation, impairment of immune system and genotoxic damage (Almeida et al. 2014, 2015, 2017; Freitas et al. 2016).

Individual LC_50_ values of acute tests with AMX and CBZ were > 100 mg/L. Therefore, based on the EC Directive 93/67 which classifies the substances according to their values of EC50/LC50, these substances are considered not harmful to aquatic organisms.

Our results confirm those reported in literature: AMX did not cause acute toxicity up to 100 mg/L in *Danio rerio* (Oliveira et al. 2013); EC_50_ value for CBZ was higher than 100 mg/L for the freshwater crustaceans *Thamnocephalus platyurus* and *Daphnia magna* at 24 h and 48 h, respectively (Kim et al. 2007; 2009). Conversely *T. fulvus* showed lower sensitivity than *D. magna* at 96 h (EC_50_ = 76.3 mg/L) (Kim et al. 2007) and the marine crustacean *Tisbe battagliai* at 48 h (LC_50_ = 59 mg/L) (Trombini et al 2016).

Since organisms in the environment are exposed to contaminants throughout their life cycle, chronic toxicity tests can provide more realistic data highlighting long-term responses by measuring various endpoints, such as survival and development, growth, and reproductive capacity (Biandolino et al. 2018; Prato et al. 2019).

*Tigriopus fulvus* presents a high environmental relevance playing a key role in the food chain. As a consequence, a delay in growth, development, and reproduction can produce changes in the population size affecting secondary production of organisms belonging to higher trophic levels feeding on them. The life cycle traits of the genus *Tigriopus* are well documented, which makes this species very suitable for long-term ecotoxicological studies (Kwok et al. 2009; Biandolino et al. 2018). Results from the present paper showed that chronic exposure of *T. fulvus* to both pharmaceuticals and their MIX did not affect survival, but they showed a negative impact to sub-lethal endpoints. The transition from the naupliar stage to the copepodite stage proved to be a sensitive endpoint. In particular, the development of *T. fulvus* exposed to AMX and CBZ after 5 days at the highest tested concentrations was delayed compared to the control, with 44 and 41% of nauplii having developed to the copepodite stage at 9.54 μg/L and 8.88 μg/L of AMX and CBZ, respectively (Fig. 1).

Chen et al. (2019a, b) showed in *Daphnia similis* and the crab *Eriocheir sinensis* the inhibition of the moulting process after exposure to CBZ by interfering with the activity of chitinolytic enzymes and moulting hormone signalling, confirming that CBZ may have long-term effects on the development.

In contrast, the exposure to the lowest concentration of AMX, CBZ, and related MIX determined stimulatory effects on the development, showing a percentage of copepodites of 82%, 73%, and 83% respectively (Fig. 1).

The moulting process is an important biological process for growth, development, and reproduction of crustaceans (Biandolino et al. 2018; Prato et al. 2019), suggesting that an alteration of the processes related to metamorphosis could be related to an impairment of the hormonal mechanisms necessary for growth (Dahl and Breitholz 2008; Subramoniam 2000). The chronic exposure to AMX and CBZ moulting and their binary combination did not affect either the first mating or the appearance of the first ovigerous female. However, a shorter time of ovigerous female appearance was observed only at the lowest concentrations in all treatments (Table 2). Similarly, Lamichhane et al. (2013) did not observe any effect of CBZ on the time of first hatch of the cladoceran crustacean *Ceriodaphia dubia* (17.5–280 μg/L; 2 weeks of exposure). Lürling et al. (2006) found that *Daphnia pulex* matured earlier when exposed to 1 µg/L of CBZ, compared to the control.

The duration of hatching time was significantly longer than the control: (i) at 0.8 μg/L for treatments with AMX and MIX at 0.94 + 1.03; (ii) only at 8.88 μg/L for CBZ.

Considering the mean number of broods per female and the total number of nauplii per female over 28 days, results showed that AMX and MIX treatments induced a similar decrease of the reproduction rate, while CBZ induced a biphasic concentration–response curve. Exposure to 0.08, 0.79, and 9.54 μg/L of CBZ resulted in a slight decrease of reproduction rate, contrary to the highest concentration (93.4 μg/L), which showed an activity pattern comparable to the control. A similar pattern was observed in the crustacean *Gammarus pulex*. The concentration–response curve shows a reduced activity (e.g., locomotion and feed frequency) at lower CBZ concentrations (10–100 ng/L) and increased at higher concentrations (1 μg/L–1 mg/L). This behaviour could be an adaptive mechanism to a stress response (De Lange et al. 2006).

Chen et al. (2019b) stated that CBZ negatively affected reproductive parameters of *Daphnia similis* at a concentration of 0.03 μg/L. A reduction of offspring was also observed at higher concentrations between 100 and 200 μg/L in *D. magna* (Oropesa et al. 2016), *D. pulex* (Lürling et al. 2006), and *Ceriodaphia dubia* (Lamichhane et al. 2013). The exposure of zebrafish between 0.5 and 10 μg/L of CBZ caused a decrease of egg production, because of a reduced stimulation of neurons resulting in a reduction of excitability in reproductive organs and synthesis of gonadal steroids (Galus et al. 2013). *Carpa carpio* showed a decreased motility and velocity of sperms after 2 h of exposure to 2000 and 20,000 μg/L of CBZ (Li et al. 2010).

Lürling et al. (2006) and Rivetti et al (2016) showed that, as a neuro-active pharmaceutical, CBZ was able to enhance reproduction at 1 μg/L on *Daphnia pulex* and *D. magna*, respectively.

With regard to AMX, there is a paucity of data on long-term toxicity studies on aquatic organisms (Park and Choi 2008). Our results agreed with González-Pérez et al. (2016) observing that AMX negatively affected the survival and reproduction of two rotifer species: *Brachionus calyciflorus* and *B. havanaensis* especially when exposed to or above 100 μg/L.

In the present study, AMX and CBZ were evaluated in a 1:1 ratio at concentrations comparable to the single substance test. The hazard of pollutant mixtures can be particularly insidious because they may interact to cause adverse effects in marine environments. Results from MATC showed that, for most of the evaluated endpoints, mixture values were lower than the action of drugs considered singly. About the number of nauplii per female, the lowest value of MATC in the mixture could be due to the antibiotic effect that probably prevailed on that of the antiepiletiptic drug (Table 2).

Aborted eggs can be also considered an interesting and sensitive endpoint. All treatments at the highest concentrations produced aborted eggs suggesting that the tested pharmaceuticals can directly impair broods. In particular, the ratio of abortion from the mixture of 1.030 μg/L of AMX and 0.941 μg/L of CBZ was like that found in the single drug exposures suggesting that AMX and CBZ could act in mixture via an additive effect.

## Conclusions

At present, there is an urgent need to prioritize pharmaceutical compounds for an appropriate environmental risk assessment in aquatic environments, especially saltwater ones, mainly due to data heterogeneity and fragmentation and the need to establish threshold limit concentrations especially for sub-lethal endpoints.

Results from the present study suggested that amoxicillin and carbamazepine did not exert acute effects even at concentrations many orders of magnitude higher than those detected in the environment, and thus, they cannot be considered dangerous to *T. fulvus.* Anyhow, reproduction-related endpoints evidenced that AMX and CBZ can exert some sub-lethal effects on *T. fulvus* even at very low concentrations (approximately 1 μg/L), including not only pure substances but also their mixtures. Declining fertility is a key aspect that could have serious ecological consequences due to the long-term exposure of aquatic organisms to the tested drugs affecting population growth. These findings suggest that the individual life cycle traits of testing species can significantly improve the ability to estimate the impact of pharmaceuticals at environmentally representative concentrations.

## Supplementary Information

Below is the link to the electronic supplementary material.

Supplementary file1 (DOCX 71 KB)

## Data Availability

All data generated or analyzed during this study are included in this published article (see Supplementary Materials).
