# Supplementary material for: Individual and combined effects of amoxicillin and carbamazepine to the marine copepod Tigriopus fulvus
Source: Environ Sci Pollut Res Int. 2023 Mar 18;30(22):61672–81. doi: 10.1007/s11356-023-26498-0 (PMC10167106; doi:10.1007/s11356-023-26498-0)
Supplement: Supplementary file 1 — Supplementary file1 (DOCX 71 KB) [file 11356_2023_26498_MOESM1_ESM.docx]

**Supplementary Materials**

Table S1 Summary of experimental conditions of each toxicity test.

|  | **Acute test** | **Chronic test** |
| --- | --- | --- |
|  |  |  |
| Test type | Semi-static | Semi-static |
| Stage of development | Nauplii (≤24h) | Nauplii (≤24h) |
|  |  |  |
| Light/dark photoperiod (h) | 16:8 | 16:8 |
|  |  |  |
| Dilution water | Artificial Sea Water (ASW)  (0.22 μm) | Artificial Sea Water (ASW)  (0.22 μm) |
|  |  |  |
| Salinity (‰) | 38 ± 2 | 38 ± 2 |
|  |  |  |
| Temperature | 20 ± 2 | 20 ± 2 |
|  |  |  |
| pH | 8 ± 0.3 | 8 ± 0.3 |
|  |  |  |
| Oxygen (%) | >80 | > 80 |
|  |  |  |
| Chamber test | 12-well plates | 12-well plates |
|  |  |  |
| Test duration | 96 h | 28 d |
|  |  |  |
| Incubation volume (mL) | 3 | 4 |
|  |  |  |
| Number of organisms per replicate | 10 | 12-13 |
|  |  |  |
| Number of replicates | 3 | 3 |
|  |  |  |
|  |  |  |
| Number of concentrations | 5 + control | 4 + control |
|  |  |  |
| Concentrations (AMX, CBZ, MIX) | 6.25, 12.5, 25, 50, 100  mg /L of AMX and CBZ  12.5 25 – 50 – 100 - 200  mg /L MIX | 0.1,1,10,100 μg /L  of AMX and CBZ  0.1 ,1,10,100  μg/L of MIX |
|  |  |  |
| Solution renewal | Every 48 h | Every 48 h |
|  |  |  |
| Feeding during testing | Absent | *Tetraselmis suecica*  10^5^ cells/L |
|  |  |  |
| Endpoint | Mortality rate | Survival, larval development, maturation time, sex-ratio, hatching time, number broods/female, number nauplii/brood, number nauplii/female, aborted sacs |
|  |  |  |
|  |  |  |

Table S2 Average time (days) necessary to females to be ovigerous being exposed to amoxicillin (AMX), carbamazepine (CBZ), and their mixture 1:1 (MIX). The concentrations are in µg/L referring to single concentrations reported in Table 1; data are in days (mean ± SD, N = 3, n = 9).

| Concentrations (µg/L) |  | AMX | |  | Concentrations (µg/L) | CBZ | |  | Concentrations (µg/L) | MIX (1:1) | |
| --- | --- | --- | --- | --- | --- | --- | --- | --- | --- | --- | --- |
|  |  |  | Mean ± s.d |  |  |  | Mean ± s.d |  |  |  | Mean ± s.d |
|  | Run 1 | 13.0 ± 0.0 |  |  |  | 13.0 ± 0.0 |  |  |  | 13.0 ± 0.0 |  |
| 0 | Run 2 | 13.8 ± 0.4 | 13.6 ± 0.5 |  | 0 | 13.8 ± 0.4 | 13.6 ± 0.5 |  | 0 | 13.8 ± 0.4 | 13.6 ± 0.5 |
|  | Run 3 | 14.0 ± 0.0 |  |  |  | 14.0 ± 0.0 |  |  |  | 14.0 ± 0.0 |  |
|  |  |  |  |  |  |  |  |  |  |  |  |
|  | Run 1 | 12.2 ± 0.4 |  |  |  | 12.0 ± 0.0 |  |  |  | 12.0 ± 0.0 |  |
| 0.08 | Run 2 | 12.0 ± 0.0 | 12.4 ± 0.5 |  | 0.16 | 12.4 ± 0.5 | 12.4 ± 0.5 |  | 0.08+0.08 | 12.0 ± 0.0 | 12.2 ± 0.4 |
|  | Run 3 | 13.0 ± 0.4 |  |  |  | 13.0 ± 0.0 |  |  |  | 12.6 ± 0.5 |  |
|  |  |  |  |  |  |  |  |  |  |  |  |
|  | Run 1 | 14.0 ± 0.0 |  |  |  | 12.8 ± 0.4 |  |  |  | 12.6 ± 0.5 |  |
| 0.79 | Run 2 | 13.2 ± 0.8 | 13.7 ± 0.5 |  | 1.26 | 13.0 ± 0.0 | 13.4 ± 0.8 |  | 0.94+1.03 | 13.0 ± 0.0 | 13.3 ± 0.9 |
|  | Run 3 | 14.0 ± 0.0 |  |  |  | 14.4 ± 0.9 |  |  |  | 14.4 ± 0.9 |  |
|  |  |  |  |  |  |  |  |  |  |  |  |
|  | Run 1 | 13.0 ± 0.0 |  |  |  | 13.4 ± 0.5 |  |  |  | 13.6 ± 0.5 |  |
| 9.54 | Run 2 | 14.2 ± 0.8 | 13.9 ± 0.8 |  | 8.88 | 14.2 ± 0.8 | 14.2 ± 0.8 |  | 9.21+8.74 | 14.0 ± 0.9 | 14.3 ± 0.7 |
|  | Run 3 | 14.6 ± 0.5 |  |  |  | 15.0 ± 0.0 |  |  |  | 15.0 ± 0.0 |  |
|  |  |  |  |  |  |  |  |  |  |  |  |
|  | Run 1 | 13.4 ± 0.5 |  |  |  | 13.2 ± 0.8 |  |  |  | 13.2 ± 0.4 |  |
| 92.3 | Run 2 | 13.8 ± 0.8 | 14.0 ± 0.8 |  | 95.6 | 14.4 ± 0.5 | 14.2 ± 0.9 |  | 96.34+94.67 | 13.8 ± 0.8 | 13.9 ± 0.9 |
|  | Run 3 | 15.0 ± 0.5 |  |  |  | 15.0 ± 0.0 |  |  |  | 15.0 ± 0.0 |  |

Table S3 Nested analysis of variance of the time (days) necessary to females to be ovigerous after the exposure to amoxicillin (AMX), carbamazepine (CBZ), and their mixture 1:1 (MIX) (see Table 3 in the main manuscript); ctr (negative control), R = replicate, Level of statistical significance: * = 0.05; ** = 0.1; *** = 0.01; **** = 0.001; ns = not significant.

| AMX | | |  | CBZ | | |  | MIX | | |
| --- | --- | --- | --- | --- | --- | --- | --- | --- | --- | --- |
| Tukey's multiple comparisons test | Summary | Adjusted P Value |  | Tukey's multiple comparisons test | Summary | Adjusted P Value |  | Tukey's multiple comparisons test | Summary | Adjusted P Value |
| ctr:R1 vs. ctr:R2 | * | 0.011 |  | ctr:R1 vs. ctr:R2 | * | 0.011 |  | ctr:R1 vs. ctr:R2 | ** | 0.0093 |
| ctr:R1 vs. ctr:R3 | *** | 0.0002 |  | ctr:R1 vs. ctr:R3 | *** | 0.0002 |  | ctr:R1 vs. ctr:R3 | *** | 0.0002 |
| ctr:R1 vs. 0.1:R1 | * | 0.011 |  | ctr:R1 vs. 0.1:R1 | *** | 0.0002 |  | ctr:R1 vs. 0.1:R1 | *** | 0.0002 |
| ctr:R1 vs. 0.1:R2 | *** | 0.0002 |  | ctr:R1 vs. 0.1:R2 | ns | 0.1895 |  | ctr:R1 vs. 0.1:R2 | *** | 0.0002 |
| ctr:R1 vs. 0.1:R3 | ns | 0.9997 |  | ctr:R1 vs. 0.1:R3 | ns | >0.9999 |  | ctr:R1 vs. 0.1:R3 | ns | 0.8012 |
| ctr:R1 vs. 1:R1 | *** | 0.0002 |  | ctr:R1 vs. 1:R1 | ns | 0.9997 |  | ctr:R1 vs. 1:R1 | ns | 0.8012 |
| ctr:R1 vs. 1:R2 | ns | 0.9997 |  | ctr:R1 vs. 1:R2 | ns | >0.9999 |  | ctr:R1 vs. 1:R2 | ns | >0.9999 |
| ctr:R1 vs. 1:R3 | *** | 0.0002 |  | ctr:R1 vs. 1:R3 | **** | <0.0001 |  | ctr:R1 vs. 1:R3 | **** | <0.0001 |
| ctr:R1 vs. 10:R1 | ns | >0.9999 |  | ctr:R1 vs. 10:R1 | ns | 0.8153 |  | ctr:R1 vs. 10:R1 | ns | 0.1739 |
| ctr:R1 vs. 10:R2 | **** | <0.0001 |  | ctr:R1 vs. 10:R2 | **** | <0.0001 |  | ctr:R1 vs. 10:R2 | **** | <0.0001 |
| ctr:R1 vs. 10:R3 | **** | <0.0001 |  | ctr:R1 vs. 10:R3 | **** | <0.0001 |  | ctr:R1 vs. 10:R3 | **** | <0.0001 |
| ctr:R1 vs. 100:R1 | ns | 0.8153 |  | ctr:R1 vs. 100:R1 | ns | 0.9997 |  | ctr:R1 vs. 100:R1 | ns | 0.9996 |
| ctr:R1 vs. 100:R2 | * | 0.011 |  | ctr:R1 vs. 100:R2 | **** | <0.0001 |  | ctr:R1 vs. 100:R2 | ** | 0.0093 |
| ctr:R1 vs. 100:R3 | **** | <0.0001 |  | ctr:R1 vs. 100:R3 | **** | <0.0001 |  | ctr:R1 vs. 100:R3 | **** | <0.0001 |
| ctr:R2 vs. ctr:R3 | ns | 0.9997 |  | ctr:R2 vs. ctr:R3 | ns | 0.9997 |  | ctr:R2 vs. ctr:R3 | ns | 0.9996 |
| ctr:R2 vs. 0.1:R1 | **** | <0.0001 |  | ctr:R2 vs. 0.1:R1 | **** | <0.0001 |  | ctr:R2 vs. 0.1:R1 | **** | <0.0001 |
| ctr:R2 vs. 0.1:R2 | **** | <0.0001 |  | ctr:R2 vs. 0.1:R2 | **** | <0.0001 |  | ctr:R2 vs. 0.1:R2 | **** | <0.0001 |
| ctr:R2 vs. 0.1:R3 | ns | 0.1895 |  | ctr:R2 vs. 0.1:R3 | * | 0.011 |  | ctr:R2 vs. 0.1:R3 | **** | <0.0001 |
| ctr:R2 vs. 1:R1 | ns | 0.9997 |  | ctr:R2 vs. 1:R1 | *** | 0.0002 |  | ctr:R2 vs. 1:R1 | **** | <0.0001 |
| ctr:R2 vs. 1:R2 | ns | 0.1895 |  | ctr:R2 vs. 1:R2 | * | 0.011 |  | ctr:R2 vs. 1:R2 | ** | 0.0093 |
| ctr:R2 vs. 1:R3 | ns | 0.9997 |  | ctr:R2 vs. 1:R3 | ns | 0.1895 |  | ctr:R2 vs. 1:R3 | ns | 0.1739 |
| ctr:R2 vs. 10:R1 | * | 0.011 |  | ctr:R2 vs. 10:R1 | ns | 0.8153 |  | ctr:R2 vs. 10:R1 | ns | 0.9996 |
| ctr:R2 vs. 10:R2 | ns | 0.8153 |  | ctr:R2 vs. 10:R2 | ns | 0.8153 |  | ctr:R2 vs. 10:R2 | ns | 0.9754 |
| ctr:R2 vs. 10:R3 | * | 0.011 |  | ctr:R2 vs. 10:R3 | **** | <0.0001 |  | ctr:R2 vs. 10:R3 | **** | <0.0001 |
| ctr:R2 vs. 100:R1 | ns | 0.8153 |  | ctr:R2 vs. 100:R1 | ns | 0.1895 |  | ctr:R2 vs. 100:R1 | ns | 0.1739 |
| ctr:R2 vs. 100:R2 | ns | >0.9999 |  | ctr:R2 vs. 100:R2 | ns | 0.1895 |  | ctr:R2 vs. 100:R2 | ns | >0.9999 |
| ctr:R2 vs. 100:R3 | **** | <0.0001 |  | ctr:R2 vs. 100:R3 | **** | <0.0001 |  | ctr:R2 vs. 100:R3 | **** | <0.0001 |
| ctr:R3 vs. 0.1:R1 | **** | <0.0001 |  | ctr:R3 vs. 0.1:R1 | **** | <0.0001 |  | ctr:R3 vs. 0.1:R1 | **** | <0.0001 |
| ctr:R3 vs. 0.1:R2 | **** | <0.0001 |  | ctr:R3 vs. 0.1:R2 | **** | <0.0001 |  | ctr:R3 vs. 0.1:R2 | **** | <0.0001 |
| ctr:R3 vs. 0.1:R3 | * | 0.011 |  | ctr:R3 vs. 0.1:R3 | *** | 0.0002 |  | ctr:R3 vs. 0.1:R3 | **** | <0.0001 |
| ctr:R3 vs. 1:R1 | ns | >0.9999 |  | ctr:R3 vs. 1:R1 | **** | <0.0001 |  | ctr:R3 vs. 1:R1 | **** | <0.0001 |
| ctr:R3 vs. 1:R2 | * | 0.011 |  | ctr:R3 vs. 1:R2 | *** | 0.0002 |  | ctr:R3 vs. 1:R2 | *** | 0.0002 |
| ctr:R3 vs. 1:R3 | ns | >0.9999 |  | ctr:R3 vs. 1:R3 | ns | 0.8153 |  | ctr:R3 vs. 1:R3 | ns | 0.8012 |
| ctr:R3 vs. 10:R1 | *** | 0.0002 |  | ctr:R3 vs. 10:R1 | ns | 0.1895 |  | ctr:R3 vs. 10:R1 | ns | 0.8012 |
| ctr:R3 vs. 10:R2 | ns | 0.9997 |  | ctr:R3 vs. 10:R2 | ns | 0.9997 |  | ctr:R3 vs. 10:R2 | ns | >0.9999 |
| ctr:R3 vs. 10:R3 | ns | 0.1895 |  | ctr:R3 vs. 10:R3 | *** | 0.0002 |  | ctr:R3 vs. 10:R3 | *** | 0.0002 |
| ctr:R3 vs. 100:R1 | ns | 0.1895 |  | ctr:R3 vs. 100:R1 | * | 0.011 |  | ctr:R3 vs. 100:R1 | ** | 0.0093 |
| ctr:R3 vs. 100:R2 | ns | 0.9997 |  | ctr:R3 vs. 100:R2 | ns | 0.8153 |  | ctr:R3 vs. 100:R2 | ns | 0.9996 |
| ctr:R3 vs. 100:R3 | **** | <0.0001 |  | ctr:R3 vs. 100:R3 | *** | 0.0002 |  | ctr:R3 vs. 100:R3 | *** | 0.0002 |
| 0.1:R1 vs. 0.1:R2 | ns | 0.9997 |  | 0.1:R1 vs. 0.1:R2 | ns | 0.8153 |  | 0.1:R1 vs. 0.1:R2 | ns | >0.9999 |
| 0.1:R1 vs. 0.1:R3 | *** | 0.0002 |  | 0.1:R1 vs. 0.1:R3 | *** | 0.0002 |  | 0.1:R1 vs. 0.1:R3 | ns | 0.1739 |
| 0.1:R1 vs. 1:R1 | **** | <0.0001 |  | 0.1:R1 vs. 1:R1 | * | 0.011 |  | 0.1:R1 vs. 1:R1 | ns | 0.1739 |
| 0.1:R1 vs. 1:R2 | *** | 0.0002 |  | 0.1:R1 vs. 1:R2 | *** | 0.0002 |  | 0.1:R1 vs. 1:R2 | *** | 0.0002 |
| 0.1:R1 vs. 1:R3 | **** | <0.0001 |  | 0.1:R1 vs. 1:R3 | **** | <0.0001 |  | 0.1:R1 vs. 1:R3 | **** | <0.0001 |
| 0.1:R1 vs. 10:R1 | * | 0.011 |  | 0.1:R1 vs. 10:R1 | **** | <0.0001 |  | 0.1:R1 vs. 10:R1 | **** | <0.0001 |
| 0.1:R1 vs. 10:R2 | **** | <0.0001 |  | 0.1:R1 vs. 10:R2 | **** | <0.0001 |  | 0.1:R1 vs. 10:R2 | **** | <0.0001 |
| 0.1:R1 vs. 10:R3 | **** | <0.0001 |  | 0.1:R1 vs. 10:R3 | **** | <0.0001 |  | 0.1:R1 vs. 10:R3 | **** | <0.0001 |
| 0.1:R1 vs. 100:R1 | **** | <0.0001 |  | 0.1:R1 vs. 100:R1 | **** | <0.0001 |  | 0.1:R1 vs. 100:R1 | **** | <0.0001 |
| 0.1:R1 vs. 100:R2 | **** | <0.0001 |  | 0.1:R1 vs. 100:R2 | **** | <0.0001 |  | 0.1:R1 vs. 100:R2 | **** | <0.0001 |
| 0.1:R1 vs. 100:R3 | **** | <0.0001 |  | 0.1:R1 vs. 100:R3 | **** | <0.0001 |  | 0.1:R1 vs. 100:R3 | **** | <0.0001 |
| 0.1:R2 vs. 0.1:R3 | **** | <0.0001 |  | 0.1:R2 vs. 0.1:R3 | ns | 0.1895 |  | 0.1:R2 vs. 0.1:R3 | ns | 0.1739 |
| 0.1:R2 vs. 1:R1 | **** | <0.0001 |  | 0.1:R2 vs. 1:R1 | ns | 0.8153 |  | 0.1:R2 vs. 1:R1 | ns | 0.1739 |
| 0.1:R2 vs. 1:R2 | **** | <0.0001 |  | 0.1:R2 vs. 1:R2 | ns | 0.1895 |  | 0.1:R2 vs. 1:R2 | *** | 0.0002 |
| 0.1:R2 vs. 1:R3 | **** | <0.0001 |  | 0.1:R2 vs. 1:R3 | **** | <0.0001 |  | 0.1:R2 vs. 1:R3 | **** | <0.0001 |
| 0.1:R2 vs. 10:R1 | *** | 0.0002 |  | 0.1:R2 vs. 10:R1 | *** | 0.0002 |  | 0.1:R2 vs. 10:R1 | **** | <0.0001 |
| 0.1:R2 vs. 10:R2 | **** | <0.0001 |  | 0.1:R2 vs. 10:R2 | **** | <0.0001 |  | 0.1:R2 vs. 10:R2 | **** | <0.0001 |
| 0.1:R2 vs. 10:R3 | **** | <0.0001 |  | 0.1:R2 vs. 10:R3 | **** | <0.0001 |  | 0.1:R2 vs. 10:R3 | **** | <0.0001 |
| 0.1:R2 vs. 100:R1 | **** | <0.0001 |  | 0.1:R2 vs. 100:R1 | * | 0.011 |  | 0.1:R2 vs. 100:R1 | **** | <0.0001 |
| 0.1:R2 vs. 100:R2 | **** | <0.0001 |  | 0.1:R2 vs. 100:R2 | **** | <0.0001 |  | 0.1:R2 vs. 100:R2 | **** | <0.0001 |
| 0.1:R2 vs. 100:R3 | **** | <0.0001 |  | 0.1:R2 vs. 100:R3 | **** | <0.0001 |  | 0.1:R2 vs. 100:R3 | **** | <0.0001 |
| 0.1:R3 vs. 1:R1 | * | 0.011 |  | 0.1:R3 vs. 1:R1 | ns | 0.9997 |  | 0.1:R3 vs. 1:R1 | ns | >0.9999 |
| 0.1:R3 vs. 1:R2 | ns | >0.9999 |  | 0.1:R3 vs. 1:R2 | ns | >0.9999 |  | 0.1:R3 vs. 1:R2 | ns | 0.8012 |
| 0.1:R3 vs. 1:R3 | * | 0.011 |  | 0.1:R3 vs. 1:R3 | **** | <0.0001 |  | 0.1:R3 vs. 1:R3 | **** | <0.0001 |
| 0.1:R3 vs. 10:R1 | ns | 0.9997 |  | 0.1:R3 vs. 10:R1 | ns | 0.8153 |  | 0.1:R3 vs. 10:R1 | *** | 0.0002 |
| 0.1:R3 vs. 10:R2 | *** | 0.0002 |  | 0.1:R3 vs. 10:R2 | **** | <0.0001 |  | 0.1:R3 vs. 10:R2 | **** | <0.0001 |
| 0.1:R3 vs. 10:R3 | **** | <0.0001 |  | 0.1:R3 vs. 10:R3 | **** | <0.0001 |  | 0.1:R3 vs. 10:R3 | **** | <0.0001 |
| 0.1:R3 vs. 100:R1 | ns | 0.9997 |  | 0.1:R3 vs. 100:R1 | ns | 0.9997 |  | 0.1:R3 vs. 100:R1 | ns | 0.1739 |
| 0.1:R3 vs. 100:R2 | ns | 0.1895 |  | 0.1:R3 vs. 100:R2 | **** | <0.0001 |  | 0.1:R3 vs. 100:R2 | **** | <0.0001 |
| 0.1:R3 vs. 100:R3 | **** | <0.0001 |  | 0.1:R3 vs. 100:R3 | **** | <0.0001 |  | 0.1:R3 vs. 100:R3 | **** | <0.0001 |
| 1:R1 vs. 1:R2 | * | 0.011 |  | 1:R1 vs. 1:R2 | ns | 0.9997 |  | 1:R1 vs. 1:R2 | ns | 0.8012 |
| 1:R1 vs. 1:R3 | ns | >0.9999 |  | 1:R1 vs. 1:R3 | **** | <0.0001 |  | 1:R1 vs. 1:R3 | **** | <0.0001 |
| 1:R1 vs. 10:R1 | *** | 0.0002 |  | 1:R1 vs. 10:R1 | ns | 0.1895 |  | 1:R1 vs. 10:R1 | *** | 0.0002 |
| 1:R1 vs. 10:R2 | ns | 0.9997 |  | 1:R1 vs. 10:R2 | **** | <0.0001 |  | 1:R1 vs. 10:R2 | **** | <0.0001 |
| 1:R1 vs. 10:R3 | ns | 0.1895 |  | 1:R1 vs. 10:R3 | **** | <0.0001 |  | 1:R1 vs. 10:R3 | **** | <0.0001 |
| 1:R1 vs. 100:R1 | ns | 0.1895 |  | 1:R1 vs. 100:R1 | ns | 0.8153 |  | 1:R1 vs. 100:R1 | ns | 0.1739 |
| 1:R1 vs. 100:R2 | ns | 0.9997 |  | 1:R1 vs. 100:R2 | **** | <0.0001 |  | 1:R1 vs. 100:R2 | **** | <0.0001 |
| 1:R1 vs. 100:R3 | **** | <0.0001 |  | 1:R1 vs. 100:R3 | **** | <0.0001 |  | 1:R1 vs. 100:R3 | **** | <0.0001 |
| 1:R2 vs. 1:R3 | * | 0.011 |  | 1:R2 vs. 1:R3 | **** | <0.0001 |  | 1:R2 vs. 1:R3 | **** | <0.0001 |
| 1:R2 vs. 10:R1 | ns | 0.9997 |  | 1:R2 vs. 10:R1 | ns | 0.8153 |  | 1:R2 vs. 10:R1 | ns | 0.1739 |
| 1:R2 vs. 10:R2 | *** | 0.0002 |  | 1:R2 vs. 10:R2 | **** | <0.0001 |  | 1:R2 vs. 10:R2 | **** | <0.0001 |
| 1:R2 vs. 10:R3 | **** | <0.0001 |  | 1:R2 vs. 10:R3 | **** | <0.0001 |  | 1:R2 vs. 10:R3 | **** | <0.0001 |
| 1:R2 vs. 100:R1 | ns | 0.9997 |  | 1:R2 vs. 100:R1 | ns | 0.9997 |  | 1:R2 vs. 100:R1 | ns | 0.9996 |
| 1:R2 vs. 100:R2 | ns | 0.1895 |  | 1:R2 vs. 100:R2 | **** | <0.0001 |  | 1:R2 vs. 100:R2 | ** | 0.0093 |
| 1:R2 vs. 100:R3 | **** | <0.0001 |  | 1:R2 vs. 100:R3 | **** | <0.0001 |  | 1:R2 vs. 100:R3 | **** | <0.0001 |
| 1:R3 vs. 10:R1 | *** | 0.0002 |  | 1:R3 vs. 10:R1 | *** | 0.0002 |  | 1:R3 vs. 10:R1 | ** | 0.0093 |
| 1:R3 vs. 10:R2 | ns | 0.9997 |  | 1:R3 vs. 10:R2 | ns | 0.9997 |  | 1:R3 vs. 10:R2 | ns | 0.9754 |
| 1:R3 vs. 10:R3 | ns | 0.1895 |  | 1:R3 vs. 10:R3 | ns | 0.1895 |  | 1:R3 vs. 10:R3 | ns | 0.1739 |
| 1:R3 vs. 100:R1 | ns | 0.1895 |  | 1:R3 vs. 100:R1 | **** | <0.0001 |  | 1:R3 vs. 100:R1 | **** | <0.0001 |
| 1:R3 vs. 100:R2 | ns | 0.9997 |  | 1:R3 vs. 100:R2 | ns | >0.9999 |  | 1:R3 vs. 100:R2 | ns | 0.1739 |
| 1:R3 vs. 100:R3 | **** | <0.0001 |  | 1:R3 vs. 100:R3 | ns | 0.1895 |  | 1:R3 vs. 100:R3 | ns | 0.1739 |
| 10:R1 vs. 10:R2 | **** | <0.0001 |  | 10:R1 vs. 10:R2 | * | 0.011 |  | 10:R1 vs. 10:R2 | ns | 0.4574 |
| 10:R1 vs. 10:R3 | **** | <0.0001 |  | 10:R1 vs. 10:R3 | **** | <0.0001 |  | 10:R1 vs. 10:R3 | **** | <0.0001 |
| 10:R1 vs. 100:R1 | ns | 0.8153 |  | 10:R1 vs. 100:R1 | ns | 0.9997 |  | 10:R1 vs. 100:R1 | ns | 0.8012 |
| 10:R1 vs. 100:R2 | * | 0.011 |  | 10:R1 vs. 100:R2 | *** | 0.0002 |  | 10:R1 vs. 100:R2 | ns | 0.9996 |
| 10:R1 vs. 100:R3 | **** | <0.0001 |  | 10:R1 vs. 100:R3 | **** | <0.0001 |  | 10:R1 vs. 100:R3 | **** | <0.0001 |
| 10:R2 vs. 10:R3 | ns | 0.8153 |  | 10:R2 vs. 10:R3 | * | 0.011 |  | 10:R2 vs. 10:R3 | ** | 0.0015 |
| 10:R2 vs. 100:R1 | * | 0.011 |  | 10:R2 vs. 100:R1 | *** | 0.0002 |  | 10:R2 vs. 100:R1 | ** | 0.0015 |
| 10:R2 vs. 100:R2 | ns | 0.8153 |  | 10:R2 vs. 100:R2 | ns | 0.9997 |  | 10:R2 vs. 100:R2 | ns | 0.9754 |
| 10:R2 vs. 100:R3 | *** | 0.0002 |  | 10:R2 vs. 100:R3 | * | 0.011 |  | 10:R2 vs. 100:R3 | ** | 0.0015 |
| 10:R3 vs. 100:R1 | **** | <0.0001 |  | 10:R3 vs. 100:R1 | **** | <0.0001 |  | 10:R3 vs. 100:R1 | **** | <0.0001 |
| 10:R3 vs. 100:R2 | * | 0.011 |  | 10:R3 vs. 100:R2 | ns | 0.1895 |  | 10:R3 vs. 100:R2 | **** | <0.0001 |
| 10:R3 vs. 100:R3 | ns | 0.1895 |  | 10:R3 vs. 100:R3 | ns | >0.9999 |  | 10:R3 vs. 100:R3 | ns | >0.9999 |
| 100:R1 vs. 100:R2 | ns | 0.8153 |  | 100:R1 vs. 100:R2 | **** | <0.0001 |  | 100:R1 vs. 100:R2 | ns | 0.1739 |
| 100:R1 vs. 100:R3 | **** | <0.0001 |  | 100:R1 vs. 100:R3 | **** | <0.0001 |  | 100:R1 vs. 100:R3 | **** | <0.0001 |
| 100:R2 vs. 100:R3 | **** | <0.0001 |  | 100:R2 vs. 100:R3 | ns | 0.1895 |  | 100:R2 vs. 100:R3 | **** | <0.0001 |

Table S4 Hatching time (days) of *T. fulvus* exposed to amoxicillin (AMX), carbamazepine (CBZ), and their mixture 1:1 (MIX). Concentrations are in μg/L and effect data are reported as mean ± standard deviation (SD) of each run test (N = 3) and replicates (n = 3).

| Concentrations (µg/L) | |  | | AMX | | Concentrations (µg/L) | | CBZ | | Concentrations (µg/L) | | MIX (1:1) | |
| --- | --- | --- | --- | --- | --- | --- | --- | --- | --- | --- | --- | --- | --- |
|  | Run 1 | | 2.60 ± 0.1 | |  | | 2.60 ± 0.1 | |  | | 2.60 ± 0.1 | |  |
| 0 | Run 2 | | 2.28 ± 0.2 | | 0 | | 2.28 ± 0.2 | | 0 | | 2.28 ± 0.2 | |  |
|  | Run 3 | | 2.41 ± 0.2 | |  | | 2.41 ± 0.2 | |  | | 2.41 ± 0.2 | |  |
|  |  | |  | |  | |  | |  | |  | |  |
|  | Run 1 | | 2.93 ± 0.2 | |  | | 2.67 ± 0.3 | |  | | 2.70 ± 0.3 | |  |
| 0.08 | Run 2 | | 2.56 ± 0.2 | | 0.16 | | 2.67 ± 0.2 | | 0.08 + 0.16 | | 2.54 ± 0.3 | |  |
|  | Run 3 | | 2.54 ± 0.1 | |  | | 2.64 ± 0.3 | |  | | 2.58 ± 0.2 | |  |
|  |  | |  | |  | |  | |  | |  | |  |
|  | Run 1 | | 2.93 ± 0.2 | |  | | 2.70 ± 0.2 | |  | | 2.93 ± 0.1 | |  |
| 0.79 | Run 2 | | 2.82 ± 0.3 | | 1.26 | | 2.85 ± 0.3 | | 1.03 + 0.94 | | 3.16 ± 0.1 | |  |
|  | Run 3 | | 2.93 ± 0.4 | |  | | 2.61 ± 0.1 | |  | | 2.76 ± 0.4 | |  |
|  |  | |  | |  | |  | |  | |  | |  |
|  | Run 1 | | 3.06 ± 0.2 | |  | | 2.89 ± 0.5 | |  | | 2.76 ± 0.1 | |  |
| 9.54 | Run 2 | | 3.23 ± 0.5 | | 8.88 | | 2.70 ± 0.4 | | 8.74 + 9.21 | | 3.10 ± 0.6 | |  |
|  | Run 3 | | 2.61 ± 0.3 | |  | | 2.82 ± 0.2 | |  | | 2.73 ± 0.2 | |  |
|  |  | |  | |  | |  | |  | |  | |  |
|  | Run 1 | | 3.20 ± 0.2 | |  | | 2.95 ± 0.3 | |  | | 3.33 ± 0.6 | |  |
| 93.4 | Run 2 | | 3.07 ± 0.5 | | 95.6 | | 2.54 ± 0.2 | | 94.67 + 96.34 | | 2.98 ± 0.3 | |  |
|  | Run 3 | | 3.41 ± 0.5 | |  | | 2.59 ± 0.1 | |  | | 2.70 ± 0.4 | |  |

Table S5 Number of broods per female (Table 5SA) and nauplii per broods (Table 5SB) of *T. fulvus* exposed to amoxicillin (AMX), carbamazepine (CBZ), and their mixture 1:1 (MIX). Concentrations are in μg/L and effect data are reported as mean ± standard deviation (SD) of each run test (N = 3) and replicates (n = 3).

| **Table S5A Number of broods per female** | | |  |  |  |  |
| --- | --- | --- | --- | --- | --- | --- |
|  | Concentrations (µg/L) | AMX | Concentrations (µg/L) | CBZ | Concentrations (µg/L) | MIX (1:1) |
| Run 1 |  | 5.4 ± 0.5 |  | 5.4 ± 0.5 |  | 5.4 ± 0.5 |
| Run 2 | 0 | 5.8 ± 0.4 | 0 | 5.8 ± 0.4 | 0 | 5.8 ± 0.4 |
| Run 3 |  | 5.5 ± 0.6 |  | 5.5 ± 0.6 |  | 5.5 ± 0.6 |
|  |  |  |  |  |  |  |
| Run 1 |  | 5.4 ± 0.5 |  | 4.6 ± 0.8 |  | 5.4 ± 0.5 |
| Run 2 | 0.08 | 6.0 ± 0.0 | 0.16 | 5.2 ± 0.4 | 0.08+0.08 | 5.0 ± 0.8 |
| Run 3 |  | 5.7 ± 0.5 |  | 5.2 ± 0.5 |  | 5.7 ± 0.5 |
|  |  |  |  |  |  |  |
| Run 1 |  | 4.4 ± 0.5 |  | 4.8 ± 0.8 |  | 4.6 ± 0.5 |
| Run 2 | 0.79 | 4.8 ± 0.8 | 1.26 | 5.0 ± 1.0 | 0.94+1.03 | 4.4 ± 0.5 |
| Run 3 |  | 4.7 ± 0.5 |  | 4.7 ± 0.5 |  | 4.5 ± 05 |
|  |  |  |  |  |  |  |
| Run 1 |  | 4.6 ± 0.9 |  | 5.0 ± 1.0 |  | 4.6 ± 0.5 |
| Run 2 | 9.54 | 4.6 ± 0.5 | 8.88 | 5.0 ± 0.7 | 9.21+8.74 | 4.8 ± 0.4 |
| Run 3 |  | 4.2 ± 0.5 |  | 4.2 ± 0.5 |  | 4.2 ± 0.5 |
|  |  |  |  |  |  |  |
| Run 1 |  | 4.2 ± 0.4 |  | 5.0 ± 0.0 |  | 3.9 ± 0.8 |
| Run 2 | 93.4 | 4.0 ± 0.8 | 95.6 | 6.0 ± 0.2 | 96.34+94.67 | 4.0 ± 0.6 |
| Run 3 |  | 4.0 ± 0.5 |  | 5.0 ± 0.6 |  | 3.8 ± 0.6 |
|  |  |  |  |  |  |  |
| **Table S5B Number of nauplii per broods** | | |  |  |  |  |
|  | Concentrations (µg/L) | AMX | Concentrations (µg/L) | CBZ | Concentrations (µg/L) | MIX (1:1) |
| Run 1 |  | 21.2 ± 5.1 |  | 21.2 ± 5.1 |  | 21.2 ± 5.1 |
| Run 2 | 0 | 20.5 ± 3.4 | 0 | 20.5 ± 3.4 | 0 | 20.5 ± 3.4 |
| Run 3 |  | 19.1 ± 3.0 |  | 19.1 ± 3.0 |  | 19.1 ± 3.0 |
|  |  |  |  |  |  |  |
| Run 1 |  | 21.0 ± 5.8 |  | 22.6 ± 6.2 |  | 18.6 ± 3.0 |
| Run 2 | 0.08 | 22.1 ± 3.9 | 0.16 | 20.9 ± 3.9 | 0.08+0.08 | 21.6 ± 4.0 |
| Run 3 |  | 22.9 ± 2.3 |  | 22.3 ± 2.7 |  | 19.3 ± 5.4 |
|  |  |  |  |  |  |  |
| Run 1 |  | 22.7 ± 4.6 |  | 21.0 ± 5.3 |  | 24.0 ± 2.3 |
| Run 2 | 0.79 | 20.7 ± 4.6 | 1.26 | 21.2 ± 3.4 | 0.94+1.03 | 20.4 ±4.4 |
| Run 3 |  | 24.1 ± 7.1 |  | 24.8 ± 7.7 |  | 20.6 ± 3.38 |
|  |  |  |  |  |  |  |
| Run 1 |  | 18.6 ± 3.1 |  | 21.0 ± 3.5 |  | 20.4 ± 4.1 |
| Run 2 | 9.54 | 20.1 ± 3.4 | 8.88 | 21.3 ± 3.5 | 9.21+8.74 | 19.5 ± 2.7 |
| Run 3 |  | 20.7 ± 5.8 |  | 24.3 ± 5.1 |  | 24.2 ± 5.8 |
|  |  |  |  |  |  |  |
| Run 1 |  | 16.9 ± 2.5 |  | 23.8 ± 2.9 |  | 21.7 ± 3.7 |
| Run 2 | 93.4 | 20.3 ± 5.0 | 95.6 | 22.8 ± 4.1 | 96.34+94.67 | 18.4 ± 5.1 |
| Run 3 |  | 20.0 ± 2.3 |  | 19.5 ± 1.4 |  | 20.0 ± 5.3 |

Table S6. Number of nauplii per female (Table S6A) and percentage of aborted sacs (%) (Table S6B) of *T. fulvus* exposed to amoxicillin (AMX), carbamazepine (CBZ), and their mixture 1:1 (MIX). Concentrations are in μg/L and effect data are reported as mean ± standard deviation (SD) of each run test (N = 3) and replicates (n = 3).

| **Table S6A Number of nauplii per female** | | |  |  |  |  |
| --- | --- | --- | --- | --- | --- | --- |
|  | Concentrations (µg/L) | AMX | Concentrations (µg/L) | CBZ | Concentrations (µg/L) | MIX (1:1) |
| Run 1 |  | 115.2 ± 21.7 |  | 115.2 ± 21.7 |  | 115.2 ± 21.7 |
| Run 2 | 0 | 118.2 ± 26.1 | 0 | 118.2 ± 26.1 | 0 | 118.2 ± 26.1 |
| Run 3 |  | 107.2 ± 25.0 |  | 107.2 ± 25.0 |  | 107.2 ± 25.0 |
|  |  |  |  |  |  |  |
| Run 1 |  | 114 8 ± 18.3 |  | 102.2 ± 24.2 |  | 101.0 ± 21.8 |
| Run 2 | 0.08 | 132.6 ± 23.6 | 0.16 | 109.8 ±15.0 | 0.08+0.08 | 110.0 ± 18.7 |
| Run 3 |  | 132.6 ± 14.9 |  | 115.2 ± 6.4 |  | 113.0 ± 16.4 |
|  |  |  |  |  |  |  |
| Run 1 |  | 99.6 ± 19.2 |  | 102.8 ± 24.5 |  | 110.4 ± 17.0 |
| Run 2 | 0.79 | 98.0 ± 27.8 | 1.26 | 107.6 ± 22.4 | 0.94+1.03 | 90.6 ± 24.1 |
| Run 3 |  | 115.2 ± 33.8 |  | 111.4 ± 23.9 |  | 96.4 ± 16.0 |
|  |  |  |  |  |  |  |
| Run 1 |  | 85.2 ± 18.9 |  | 106.2 ± 15.3 |  | 93.4 ± 19.3 |
| Run 2 | 9.54 | 91.4 ± 9.34 | 8.88 | 105.8 ± 18.0 | 9.21+8.74 | 93.0 ± 11.0 |
| Run 3 |  | 85.8 ± 21.0 |  | 100.6 ± 15.0 |  | 101.2 ±13.7 |
|  |  |  |  |  |  |  |
| Run 1 |  | 71.4 ± 14.1 |  | 119.2 ± 14.6 |  | 81.6 ± 18.3 |
| Run 2 | 93.4 | 72.0 ±15.0 | 95.6 | 137.0 ± 24.7 | 96.34+94.67 | 82.6 ± 12.9 |
| Run 3 |  | 79.2 ± 9.8 |  | 93.6 ± 11.0 |  | 77.6 ± 8.3 |
| **Table S6B Aborted sacs percentage** | | |  |  |  |  |
|  | Concentrations (µg/L) | AMX | Concentrations (µg/L) | CBZ | Concentrations (µg/L) | MIX (1:1) |
| Run 1 |  | 0.0 ± 0.0 |  | 0.0 ± 0.0 |  | 0.0 ± 0.0 |
| Run 2 | 0 | 2.4 ± 5.8 | 0 | 2.4 ± 5.8 | 0 | 2.4 ± 5.8 |
| Run 3 |  | 2.4 ± 5.8 |  | 2.4 ± 5.8 |  | 2.4 ± 5.8 |
|  |  |  |  |  |  |  |
| Run 1 |  | 2.9 ± 7.0 |  | 2.4 ± 5.8 |  | 0.0 ± 0.0 |
| Run 2 | 0.08 | 2.4 ± 5.8 | 0.16 | 2.9 ± 7.0 | 0.08+0.08 | 4.8 ± 7.5 |
| Run 3 |  | 4.8 ± 7.5 |  | 5.2 ± 8.3 |  | 0.0 ± 0.0 |
|  |  |  |  |  |  |  |
| Run 1 |  | 0.0 ± 0.0 |  | 3.6 ± 8.7 |  | 3.6 ± 8.7 |
| Run 2 | 0.79 | 2.9 ± 7.0 | 1.26 | 2.9 ± 7.0 | 0.94+1.03 | 2.9 ± 7.0 |
| Run 3 |  | 3.6 ± 8.7 |  | 0.0 ± 0.0 |  | 3.6 ± 8.7 |
|  |  |  |  |  |  |  |
| Run 1 |  | 2.9 ± 7.0 |  | 3.6 ± 8.7 |  | 10.0 ± 11.6 |
| Run 2 | 9.54 | 3.6 ± 8.7 | 8.88 | 3.6 ± 8.7 | 9.21+8.74 | 7.1 ± 11.3 |
| Run 3 |  | 3.6 ± 8.7 |  | 0.0 ± 0.0 |  | 6.4 ± 10.2 |
|  |  |  |  |  |  |  |
| Run 1 |  | 0.0 ± 0.0 |  | 5.7 ± 9.0 |  | 11.2 ± 13.4 |
| Run 2 | 93.4 | 8.3 ± 13.4 | 95.6 | 2.9 ± 7.0 | 96.34+94.67 | 11.4± 14.4 |
| Run 3 |  | 8.3 ± 13.4 |  | 7.6 ± 12.0 |  | 11.2 ± 16.4 |
